# Clinical implication of centrosome amplification and expression of centrosomal functional genes in multiple myeloma

**DOI:** 10.1186/1479-5876-11-77

**Published:** 2013-03-23

**Authors:** Elena Dementyeva, Fedor Kryukov, Lenka Kubiczkova, Pavel Nemec, Sabina Sevcikova, Ivana Ihnatova, Jiri Jarkovsky, Jiri Minarik, Zdena Stefanikova, Petr Kuglik, Roman Hajek

**Affiliations:** 1 Babak Myeloma Group, Department of Pathological Physiology, Faculty of Medicine, Masaryk University, Kamenice 5/A3, Brno, 62500, Czech Republic; 2 Institute for Biostatistics and Analyses, Faculty of Medicine, Masaryk University, Brno, Czech Republic; 3 Department of Internal Medicine, University Hospital Olomouc, Olomouc, Czech Republic; 4 Department of Hematology and Blood Transfusion, University Hospital Bratislava, Bratislava, Slovak Republic; 5 Integrated Laboratory of Molecular Cytogenetics, Department of Experimental Biology, Faculty of Science, Masaryk University, Brno, Czech Republic

**Keywords:** Multiple myeloma, Centrosome amplification, Overall survival

## Abstract

**Background:**

Multiple myeloma (MM) is a low proliferative tumor of postgerminal center plasma cell (PC). Centrosome amplification (CA) is supposed to be one of the mechanisms leading to chromosomal instability. Also, CA is associated with deregulation of cell cycle, mitosis, DNA repair and proliferation. The aim of our study was to evaluate the prognostic significance and possible role of CA in pathogenesis and analysis of mitotic genes as mitotic disruption markers.

**Design and methods:**

A total of 173 patients were evaluated for this study. CD138+ cells were separated by MACS. Immunofluorescent labeling of centrin was used for evaluation of centrosome amplification in PCs. Interphase FISH with cytoplasmic immunoglobulin light chain staining (cIg FISH) and qRT-PCR were performed on PCs.

**Results:**

Based on the immunofluorescent staining results, all patients were divided into two groups: CA positive (38.2%) and CA negative (61.8%). Among the newly diagnosed patients, worse overall survival was indicated in the CA negative group (44/74) in comparison to the CA positive group (30/74) (*P* = 0.019).

Gene expression was significantly down-regulated in the CA positive group in comparison to CA negative in the following genes: *AURKB, PLK4, TUBG1* (*P* < 0.05). Gene expression was significantly down-regulated in newly diagnosed in comparison to relapsed patients in the following genes: *AURKA, AURKB, CCNB1, CCNB2, CETN2, HMMR, PLK4, PCNT,* and *TACC3* (*P* < 0.05).

**Conclusions:**

Our findings indicate better prognosis for CA positive newly diagnosed patients. Considering revealed clinical and gene expression heterogeneity between CA negative and CA positive patients, there is a possibility to characterize centrosome amplification as a notable event in multiple myeloma pathogenesis.

## Introduction

Centrosomes are small organelles composed of two cylindrically shaped centrioles surrounded by pericentriolar material in a normal mitotic cell. The centrosome function is to direct mitotic bipolar spindles in a process that is essential for accurate chromosome segregation during mitosis [1,2]. After the initial assumption that amplified centrosomes and abnormal mitotic arrangements might be a cause of cancer, made at the beginning of the last century, enormous efforts have been undertaken to clarify the relevance of centrosome amplification in carcinogenesis [3].

A vast number of solid and hematologic cancers harbor centrosome amplification [3]; most of them are characterized by an inherent instability of their genome [4-8]. Analyses of human tumors have revealed a strong positive correlation between centrosomal abnormalities and chromosome number aberrations [9-15] and thereupon centrosomal abnormalities are supposed to represent a poor prognostic factor. However, correlative evidence does not establish causality, and so far it has not been possible to directly show that centrosome abnormalities constitute a frequent primary cause of aneuploidy.

Conversely, there is evidence that the centrosome contributes to cell-cycle regulation and checkpoints [4,16-20]. Both centrosome duplication and DNA replication are spatially and temporally tightly linked to the cell cycle. The centrosome duplicates once every cell cycle, which starts during the G1-S transition, coincident with the onset of DNA replication. During mitosis, duplicated centrosomes direct formation of bipolar spindles, which is critical for accurate segregation of chromosomes and cytokinesis [21,22].

MM is a low proliferative tumor of postgerminal center PC. Normal PCs are arrested in the G1 phase of the cell cycle. Malignant PCs have a proliferation rate that increases from the early to the advanced stage of plasma cell dyscrasia [23-25] and this proliferative feature is one of the strongest adverse prognostic factors [25-29]. High proliferation myeloma cell index estimates range from 1 to 3% [26,30], but approximately one third of PCs contain centrosome amplification (CA) [31,32]. It appears to be plausible that deregulation of the cell cycle earlier during MM pathogenesis leads to accumulation of centrosomes and their succession to aberrant PC. Aborted PC mitoses might also contribute to causing numerical centrosome aberrations in MM [4]. In connection to the low amount of complete mitosis, CA possibly does not contribute much to accumulation of chromosome instability (CIN) in new cell generations.

Previously published data about clinical implication of CA was reported based on the so-called centosome index (CI) [31]. In fact, CI is surrogate gene expression–based index calculated by adding the normalized expression value of centrin, pericentrin and γ-tubulin genes. It is established that centrosome index correlates with CA, poor prognostic features as well as very short survival [31,33].

Although clinical implication of centrosome amplification in MM is still unclear, it is obvious that the role of CA as well as CA formation in MM differ from other malignancies. We anticipate that such differences are based on specificity of malignant transformation in MM genesis. The aim of our study was to evaluate prognostic significance and possible role of centrosome amplification in pathogenesis and analysis of mitotic genes as mitotic disruption markers.

## Design and methods

### Patients & sample preparation

A total of 173 MM patients enrolled in University Hospital Brno, Czech Republic, University Hospital Olomouc, Czech Republic and University Hospital Bratislava, Slovakia, were included in this study. The patients’ baseline characteristics are summarized in Table 1. The study was approved by the Ethical Committee of the Faculty of Medicine, Masaryk University (chairman: Josef Kure, PhD; ref number: 14/2009), and the study was conducted according to the Helsinki declaration. The bone marrow of patients was obtained during routine diagnostic procedure. Plasma cells in mononuclear cell fraction were enriched by anti-CD138+ immunomagnetic beads and sorted using AutoMACS Pro (Miltenyi Biotec). Purity of CD138+ fraction was measured by flowcytometry and/or cytospin, and samples with >80% plasma cells were provided for total RNA isolation.

**Table 1:** Patients baseline characteristics

No. of patients	173
Follow-up median (min-max) [months]	27.5 (0.4-200.8)
Gender: males-females	37.5%-62.5%
Age median (range) [years]	64 (30–84)
ISS stage: I-II-III	30.3%-31.9%-37.8%
Durie-Salmon stage: I-II-III	6.5%-14.6%-78.9%
Durie-Salmon substage: A-B	79.8%-20.2%
Ig isotype: IgG-IgA-IgM-FLC-NonSecr.	62.3%-23.6%-0.9%-12.3%-0.9%
Light chains: kappa-lambda	66%-34%
**No. of previous treatment lines**	
*None (First line treatment)*	*79 (56.0%)*
*One*	*18 (12.8%)*
*Two*	*16 (11.3%)*
*More (>2)*	*28 (19.9%)*
**Treatment regimen**	
*Bortezomib*	*50 (43.1%)*
*Thalidomid*	*40 (34.5%)*
*Lenalidomide*	*26 (22.4%)*
**Biochemical parameter**	
*Haemoglobin (g/l)*	*103.5 (73–157)*
*Thrombocytes (count x109)*	*193 (33–416)*
*Calcium (mmol/l)*	*2.3 (0.0-13.9)*
*Albumin (g/l)*	*38.3 (3.76-50.4)*
*Creatinine (umol/l)*	*101 (11–932)*
*β2-microglobulin (mg/l)*	*4.07 (1.1-42.6)*
*Lactate dehydrogenase (ukat/l)*	*3.39 (1.71-30.89)*
*C-reactive protein (mg/l)*	*4.15(0–174.3)*
*Monoclonal Ig (g/l)*	*31.05 (0–95.6)*
*Plasma cell infiltration of bonemarrow (%)*	*32.8 (1.6-93.6)*
**Chromosomal abnormality**	
*13q14 deletion*	*75 (55.1%)*
*17p13 deletion*	*19 (13.6%)*
*Translocation t(4;14)*	*18 (14.3%)*
*1q21 gain*	*64 (46.7%)*
*Hyperdiploidy*	*55 (42.0%)*

### Centrin immunofluorescent labeling and image analysis

**Figure 1:**
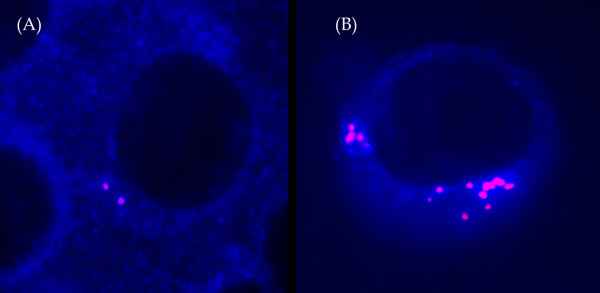
**Different pattern of centrin staining.** The isotypic PCs were identified by cytoplasmic or light chain antibody conjugated with AMCA (cIg, blue), and centrin was stained with anticentrin1/2 conjugated with TR. The cells were visualized using Olympus BX-61 fluorescent microscope with Vosskuhler 1300D digital camera and LUCIA-KARYO/FISH/CGH digital analysis system (Laboratory Imaging, s.r.o, Prague, Czech Republic). (For interpretation of the references to color in this figure (**A**) Two signals – cells with 1–4 signals were considered to have normal centrosome. (**B**) Abnormal PCs with centrosome amplification (>4 fluorescence signals of centrin).

PCs visualization with immunoglobulin light chain staining (cIg) and immunofluorescent labeling of centrin as an integral centrosome protein for centrosome copy number determination, was performed as described previously [32,34]. Brief workflow was as follows:

Cytospin slides for immunolabeling detection of CA in PCs are prepared as follows: approximately 100,000 BMMC are placed on a slide and air dried for 24 hrs at room temperature. Then, PCs are fixed in ice-cold methanol for 5 min at room temperature. Cytoplasmic membrane permeabilization is done by Triton X-100 0.2% (Affymetrix/USB, UK) for 1 minute at 37°C. After that, slides are placed in PBS for 10 min using gentle agitation. To prevent non-specific binding, blocking buffer (PBS with 10% normal goat serum – Santa Cruz Biotechnology, Inc., CA, USA) is added to each slide and incubated for 20 min in the wet chamber at 37°C. After incubation, the blocking buffer is poured off by gentle agitation during 10 min. After that, 20 μl of diluted (3 : 1000) primary antibody (Centrin 1/2 rabbit polyclonal antibody Santa Cruz Biotechnology, Inc.) is applied to each slide. The slides are incubated for 1 hr in the wet chamber at 37°C. To wash out unspecific antibody binding, slides are washed 3 times with phosphate buffered saline (PBS) for 5 min each using gentle agitation. The secondary goat anti-rabbit IgG antibody (1.5 : 1000) is applied (Santa Cruz Biotechnology, Inc.) and incubated under the same conditions for 45 min. Another step of washing is done again 3 times (PBS for 5 min, light-protected). Further immunolabeling on cytospin slides is done, using immunoglobulin light chain staining, according to the procedure described previously [7]. A cover slip is applied on all slides using mounting medium antifade without propidium iodide (PI) for PC visualization. One hundred cells were scored per each slide. Up to four centrin signals (representing four centrioles of two centrosomes) can be present in a normal cell depending on the phase of cell cycle. Samples with more than 10% of PCs with >4 signals of centrin were considered as CA-positive (Figure 1) [31,32,34].

### Fluorescence in situ hybridization (FISH)

FISH was performed as a part of routine diagnostic procedure according to the previously described protocol [35]. The following aberrations were studied: 1q21 gain, 13q14 deletion, 17p13 deletion and translocation t(4;14). The following probes were used: LSI 13q14 (RB1) Spectrum Orange Probe, LSI p53 (17p13.1) Spectrum Orange Probe, LSI IGH/FGFR3 Dual Color Probe, LSI 13q34 Spectrum Green and CEP 17 Spectrum Green reference probes (Abbott Vysis, Prague, Czech Republic). Gain 1q21 was detected using BAC DNA RP11-205 M9 probe. Hyperdiploidy status was determined with commercial probes mapping to chromosome 5 (LSI D5S23/D5S721), 9 (CEP9) and 15 (CEP15) (Abbott Molecular, Des Plaines, IL, USA). Fifty to one hundred plasma cells were evaluated for each sample. We used cut-off values recommended by the European Myeloma Network [15]. An Olympus BX 61 (Olympus, Prague, Czech Republic) fluorescence microscope and a Vosskuhler 1300D CCD camera were used for image acquisition. Image analysis was carried out using LUCIA-KARYO/FISH software (Laboratory Imaging, Prague, Czech Republic).

### RNA isolation

Total RNA was isolated using QIAGEN RNeasy Mini Kit. RNA isolation and purification were described previously [36]. RNA was isolated from either fresh or frozen material according to the manufacturer’s protocol. For frozen material, the cells were left in RLT buffer and β-mercaptoethanol for 1 hr at room temperature with occasional vortexing based on technical support information of the manufacturer to improve RNA purity.

Total RNA with purity ratio 260/280 >1.7 and integrity (RIN) >7.5 (as measured by Agilent 2010 Bionalyzer) was used for further analyses.

### Quantitative real-time PCR (qRT-PCR)

Quantitative RT-PCR was focused on a chosen list of genes, according to their role in normal centrosome duplication process. qRT-PCR was performed using the Applied Biosystems platform. Input of 100 ng of high-quality total RNA was reverse transcribed using High Capacity cDNA Reverse Transription Kit and preamplified with TaqMan PreAmp MasterMix Kit (Applied Biosystems, Foster City, CA). Expression of each gene was evaluated in a duplex reaction by TaqMan Gene Expression Assays and GAPDH as an internal control on 7500 Real Time PCR System. Relative fold change of expression for each gene was calculated using the ΔΔCt method.

### Statistical analysis

For continuous variables, nonparametric Kruskal-Wallis or Mann–Whitney tests were used. For discrete variables, chi-squared test for independence of Fisher’s exact test was used. The overall survival (OS) calculated from the date of diagnosis and survival rates were estimated using the Kaplan-Meier method. Differences of survival among subgroups of patients were compared using the log-rank test. Cox proportional hazards models were used to assess the association of several prognostic factors with OS. *P*-values below 0.05 were considered statistically significant.

## Results

### Characteristics and clinical outcome of patients

All patients involved in this study were stratified into two subgroups (CA negative and CA positive) based on centrin immunofluorescent staining results. Frequencies of patients among subgroups were as follows: 38.2% (66/173) in CA positive, 61.8% (107/173) in CA negative, respectively.

There were no significant differences regarding the presence/absence of selected chromosomal aberrations including 13q14 deletion, 17p13 deletion, translocation t(4;14), translocation t(14;16), 1q21 gain and hyperdiploidy detected by FISH in these two subgroups (Additional file 1: Table S2). Also, CA-based patient subgroups have similar distribution of Durie-Salmon staging (*P* = 0.29) as well as ISS staging (*P* = 0.46). Comparison of biochemical parameters showed significant differences in hemoglobin and creatinine level between CA positive and CA negative groups (Table 2). These differences are probably associated with the frequency of renal failure in CA positive (6/27) and CA negative group (15/44).

**Table 2:** Comparison of levels of biochemical parameters in CA groups of patients

**Biochemical parameter**	**CA negative**	**CA positive**	***P***
Hemoglobin (g/l)	99.75 (73–157)	109 (83–148)	***0.038***
Thrombocytes (count x10^9^)	199.5 (45–391)	232 (75–416)	*0.057*
Calcium (mmol/l)	2.41 (1.95-3.59)	2.29 (0.0-3.11)	*0.057*
Albumin (g/l)	35.85 (21.1-50.4)	37.4 (3.76-47.1)	*0.526*
Creatinine (umol/l)	115 (59–932)	93 (55–884)	***0.033***
β2-microglobulin (mg/l)	5.43 (1.1-42.6)	4.0 (1.36-38.2)	*0.286*
Lactate dehydrogenase (ukat/l)	3.17 (1.77-16.49)	2.95 (1.71-5.48)	*0.373*
C-reactive protein (mg/l)	5.2 (0.0-174.3)	4.0 (0.0-108.7)	*0.128*
Monoclonal Ig (g/l)	32.4 (0.0-83)	32.3 (0.0-92.6)	*0.525*
PC infiltration of bone marrow (%)	44.4 (2.2-93.6)	31.6 (4.4-82.8)	*0.432*

All values presented as median (range).

**Figure 2:**
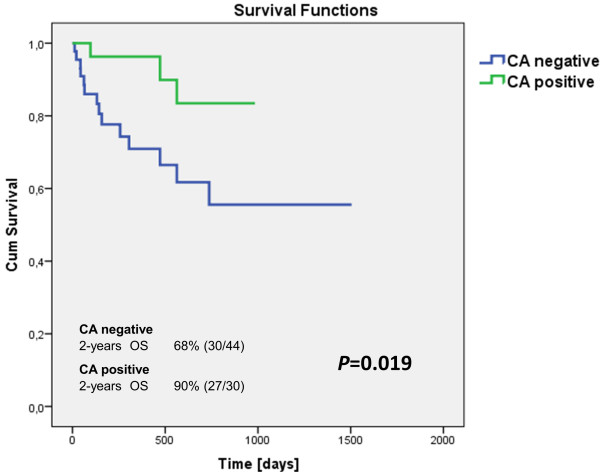
**Overall survival of CA groups of newly diagnosed MM patients.** Kaplan-Meier curves for OS of newly diagnosed MM patients (n = 74). CA positive patients (n = 30) had significantly better survival when compared to CA negative patients (n = 44) subgroups (*P* = 0.019).

To determine the prognostic impact of patients in defined subgroups, we compared their overall survival. Although statistical significance was not reached for the whole studied cohort (data not shown), significant differences were observed in newly diagnosed patients (74/139). Worse OS was indicated in CA negative patients (44/74) in comparison with CA positive patients (30/74) (*P* = 0.019) (Figure 2). None of the patients from the subgroup with prognostic implication of CA (newly diagnosed group) had transplants before sample withdrawing. But some of the patients (18/74) had transplants in further treatment lines during follow-up. Comparison of transplantation frequency between CA positive/negative groups showed an absence of significant difference (*P* > 0.05). Univariate Cox proportional hazards survival model with one explanatory variable showed worse prognosis for CA negative group (HR 0.236 [HR95%CI: 0.068; 0.818]; *P* = 0.023). All disease-related deaths occurred in two years after the diagnosis and were significantly higher in the CA negative group (7/44) than in the CA positive (0/30) group of patients (*P* = 0.037).

To further characterize the prognostic significance of CA multivariate Cox proportional hazards survival model was used (Table 3). The variables in the multivariate model were the only variables which remained statistically significant when potential predictors were combined together as well as CA which was forced into the model (Additional file 2: Table S1). The results suggest a trend of CA importance for survival of patients in spite of its border statistical significance even when combined with other predictors (Table 3).

**Table 3:** Multivariate Cox proportional hazards survival model containing centrosome amplification and other significant variables

	**HR**	**HR 95% CI**	***P***
**CA** negative	1.000		
**CA** positive	0.221	(0.049; 1.008)	0.051
**Age at time of diagnosis**	1.088	(1.023; 1.158)	**0.007**
**LDH**	1.249	(1.100; 1.419)	**0.001**

Event of interest is death (‘related to disease’ or ‘other death reason’).

### Expression level of selected genes in different subgroups of patients and gene expression-based assessment of proliferation

**Figure 3:**
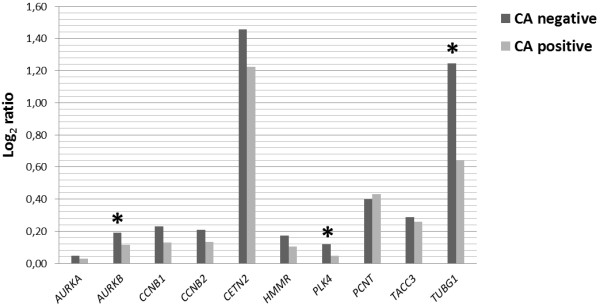
**Gene expressions in CA groups of newly diagnosed MM patients.** The expression levels of selected genes were compared in CA positive and CA negative groups of newly diagnosed MM patients. Significant differences (P < 0.05) in relative quantification coefficient R (abscissa axis) are marked with an asterisk.

In total, 10 genes (*AURKA, AURKB, CCNB1, CCNB2, HMMR, PLK4, PCNT, TUBG1, TACC3,* and *CDK2*) associated with centrosome structure/function were selected for qRT-PCR analyses. We found significant differences in relative quantification coefficient R of *AURKB, PLK4* and *TUBG1* genes that were down-regulated in the CA positive group in comparison with CA negative patients (p < 0.05) (Figure 3).

**Figure 4:**
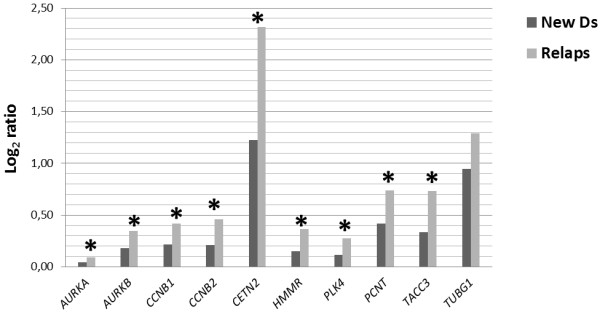
**Gene expressions in newly diagnosed and relapsed MM patients.** The expression levels of selected genes were compared in newly diagnosed and relapsed MM patients. Significant differences (P < 0.05) in relative quantification coefficient R (abscissa axis) are marked with an asterisk.

Analyses of gene expression in newly diagnosed and relapsed patients showed significant changes of the following genes: *AURKA, AURKB, CCNB1, CCNB2, CETN2, HMMR, PLK4, PCNT,* and *TACC3;* these were up-regulated in relapsed patients (p < 0.05) (Figure 4).

**Figure 5:**
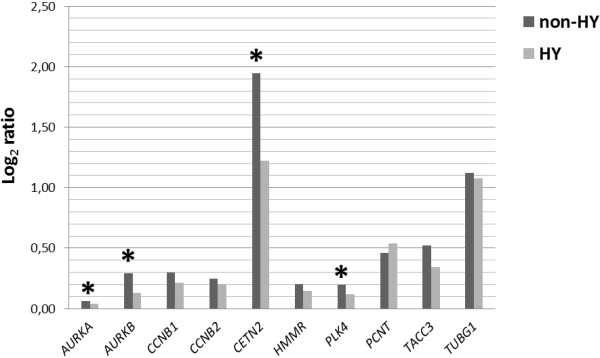
**Gene expressions in hyperdiploid and non-hyperploid MM patients.** The expression levels of selected genes were compared in hyperdiploid and non-hyperploid MM patients. Significant differences (P < 0.05) in relative quantification coefficient R (abscissa axis) are marked with an asterisk.

Expression of some genes connected with centrosome formation and function (*AURKA, AURKB, CETN2,* and *PLK4*) was significantly different between hyperdiploid and non-hyperdiploid myeloma patients (Figure 5).

## Discussion

Recent studies revealed the presence of CA in plasma cells in all stages of monoclonal gammopathies [31]. However, strong causality between CA and chromosome instability or proliferation index in MM was not shown; so far, the role of CA in MM pathogenesis is still unclear. Moreover, there is not enough information regarding the mechanism of cell cycle dysregulation in MM pathogenesis. The primary objective of this work was to evaluate the prognostic significance and possible role of centrosome amplification in pathogenesis and analysis of mitotic genes as mitotic disruption markers.

In this study, we have shown that better OS was indicated for CA positive patients. In addition, CA as a prognostic factor was relevant for disease related death cases that occurred within two years after the diagnosis.

At first sight our results contradict previously published findings. Chng et al. have shown that gene expression-based centrosome index (calculated by adding the normalized expression of three major centrosome structural proteins and components of the pericentriolar material) is associated with poor prognostic genetic subtypes and portends short survival [31,33]. On the one hand, this study has not found any association between the presence of CA (immunofluoriscent staining of centrin) and overall survival; on the other hand, this seeming contradiction can be caused by different treatment protocols used in our studies. This is determined by the year the research was conducted in. In the study of Chng and colleagues, treatment regimens were based mainly on old generation drugs (dexamethasone-based treatment or melphalan and prednisolone treatment); in the current study new treatment protocols were used (thalidomid-, bortezomib- or lenalidomide-based protocols). There is a possibility that CA-positive MM subclone is more sensitive to new agents but this anticipation needs to be proved in future detailed studies (personal communication with prof. Chng).

We suggest that CA presence can be explained as heritage from myeloma progenitors and their mitotic checkpoint alteration, whereas gene expression changes can occur in numerous physiological and/or pathological processes. This statement is in concordance with other findings in expression changes of genes associated with centrosome structure/function. Expression of the above mentioned genes was different not only in CA subgroups or related to ploid category but it was also different in newly diagnosed and relapsed patients.

A cancer cell may be derived from a mature progenitor cell that has acquired stem cell properties, i.e. self-renewal and immortality. In this case, the oncogenic event(s) enable(s) the cell(s) to self-renew. It is possible that some events, such as oncogenic targeting of the centrosome, trigger proliferation, survival and self-renewal at the same time and can target indifferently committed progenitors [37].

Although most of the multipolar divisions that occur in tumors probably reflect non-productive events, an occasional division might give rise to progeny with a genetic constitution that favors survival in a changing physiological environment. Selective pressure might arise, for instance, through increasing hypoxia or nutritional deprivation in a growing tumor mass, or through the presence of a chemotherapeutic drug [4]. Damage of G2/M and G1/S checkpoints finally provides the proliferating clone with a more aggressive armament, which includes growth factor and stromal independence, absence of response to differentiation signals and absence of polarity. It was shown by Sato et al. that centrosome overduplication may be a critical event leading to mitotic failure and subsequent cell death following crucial damaging influence and that it represents a mechanism defending the organism from abnormal cell accumulation [38]. Thus, our findings indicating better prognosis for CA positive patients are in concordance with the fact that mitotic aberrations associated with numerical and functional abnormalities of centrosomes trigger spindle checkpoints, leading to mitotic catastrophe and cell death [39]. Afterwards, because of clone selection, CA lost their positive clinical implication in cohorts of relapsed patients.

Obviously, our findings need to be confirmed and validated on a larger external cohort of patients. We believe that future investigation of centrosome amplification will help to refine the broad prognoses offered by current established systems and even sub-stratify them.

## Competing interests

The authors reported no potential competing interest.

## Authors’ contribution

ED and FK were the principal investigators and take primary responsibility for the paper. RH, JM and ZS recruited the patients. ED, FK, LK, PN, and SS performed the laboratory work for this study. II and JJ participated in the statistical analysis, RH and PK coordinated the research. ED and FK wrote the paper. All authors read and approved the final manuscript.

## Supplementary Material

Additional file 1: Table S2 Comparison of incidence of chromosomal abnormality in CA groups of patients. Click here for file

Additional file 2: Table S1 Cox proportional hazards survival model with combination of centrosome amplification and one another explanatory variable. Click here for file
