# Supplementary material for: Clinical implication of centrosome amplification and expression of centrosomal functional genes in multiple myeloma
Source: J Transl Med. 2013 Mar 23;11:77. doi: 10.1186/1479-5876-11-77 (PMC3615957; doi:10.1186/1479-5876-11-77)
Supplement: Additional file 1: Table S2 — Comparison of incidence of chromosomal abnormality in CA groups of patients. [file 1479-5876-11-77-S1.doc]

**Additional file 1: Table 2 Comparison of incidence of chromosomal abnormality in CA groups of patients**

|  |  | **CA negative** | **CA positive** | ***P*** |
| --- | --- | --- | --- | --- |
| **13q14 deletion** | Negative | 23.52% | 36.76% | 0.11 |
|  | Positive | 21.32% | 18.38% |  |
| **17p13 deletion** | Negative | 49.29% | 10.71% | 0.82 |
|  | Positive | 37.14% | 2.86% |  |
| **Translocation t(4;14)** | Negative | 52.38% | 9.52% | 0.79 |
|  | Positive | 33.33% | 4.76% |  |
| **1q21 gain** | Negative | 28.47% | 31.39% | 0.12 |
|  | Positive | 24.82% | 15.33% |  |
| **Hyperdiploidy** | Negative | 32.82% | 27.48% | 0.37 |
|  | Positive | 25.19% | 14.50% |  |
