# Supplementary material for: Clinical implication of centrosome amplification and expression of centrosomal functional genes in multiple myeloma
Source: J Transl Med. 2013 Mar 23;11:77. doi: 10.1186/1479-5876-11-77 (PMC3615957; doi:10.1186/1479-5876-11-77)
Supplement: Additional file 2: Table S1 — Cox proportional hazards survival model with combination of centrosome amplification and one another explanatory variable. [file 1479-5876-11-77-S2.doc]

**Additional file 2: Table S1. Cox proportional hazards survival model with combination of centrosome amplification and one another explanatory variable**

|  | **HR** | **HR 95% CI** | ***P*** |
| --- | --- | --- | --- |
| **CA** negative | 1.000 |  |  |
| **CA** positive | 0.199 | (0.057; 0.696) | ***0.011*** |
| **Age in time of diagnosis** | 1.088 | (1.030; 1.149) | ***0.003*** |
| **CA** negative | 1.000 |  |  |
| **CA** positive | 0.233 | (0.067; 0.813) | ***0.022*** |
| **Sex** - female | 1.000 |  |  |
| **Sex** - male | 0.907 | (0.337; 2.440) | *0.846* |
| **CA** negative | 1.000 |  |  |
| **CA** positive | 0.171 | (0.035; 0.832) | ***0.029*** |
| **D-S stage** I | 1.000 |  |  |
| **D-S stage** II | 0.631 | (0.055; 7.174) | *0.710* |
| **D-S stage** III | 0.503 | (0.107; 2.371) | *0.385* |
| **CA** negative | 1.000 |  |  |
| **CA** positive | 0.199 | (0.045; 0.880) | ***0.033*** |
| **DS substage** A | 1.000 |  |  |
| **DS substage** B | 3.341 | (1.215; 9.184) | ***0.019*** |
| **CA** negative | 1.000 |  |  |
| **CA** positive | 0.188 | (0.042; 0.846) | ***0.029*** |
| **ISS Stage** I | 1.000 |  |  |
| **ISS Stage** II | 1.020 | (0.111; 9.401) | *0.986* |
| **ISS Stage** III | 4.175 | (0.531; 32.792) | *0.174* |
| **CA** negative | 1.000 |  |  |
| **CA** positive | 0.224 | (0.050; 1.009) | *0.051* |
| **Ig isotype** - IgG | 1.000 |  |  |
| **Ig isotype** - IgA | 2.467 | (0.750; 8.121) | *0.137* |
| **Ig isotype** - LC only | 4.124 | (0.967; 17.588) | *0.056* |
| **Ig isotype** - Nonsecretory | - | - | *-* |
| **CA** negative | 1.000 |  |  |
| **CA** positive | 0.201 | (0.044; 0.909) | ***0.037*** |
| **IgL** - kappa | 1.000 |  |  |
| **IgL** - lambda | 2.666 | (0.895; 7.939) | *0.078* |
|  |  |  |  |
| **CA** negative | 1.000 |  |  |
| **CA** positive | 0.221 | (0.049; 1.000) | *0.050* |
| **Hemoglobine** | 0.983 | (0.953; 1.014) | *0.274* |
| **CA** negative | 1.000 |  |  |
| **CA** positive | 0.223 | (0.050; 0.995) | ***0.049*** |
| **Thrombocytes** | 0.994 | (0.987; 1.001) | *0.096* |
|  |  |  |  |
| **CA** negative | 1.000 |  |  |
| **CA** positive | 0.213 | (0.047; 0.961) | ***0.044*** |
| **Calcium** | 2.218 | (0.696; 7.067) | *0.178* |
| **CA** negative | 1.000 |  |  |
| **CA** positive | 0.162 | (0.035; 0.743) | ***0.019*** |
| **Albumin** | 0.953 | (0.897; 1.013) | *0.125* |
| **CA** negative | 1.000 |  |  |
| **CA** positive | 0.177 | (0.039; 0.793) | ***0.024*** |
| **Creatinine** | 1.002 | (1.000; 1.004) | *0.085* |
| **CA** negative | 1.000 |  |  |
| **CA** positive | 0.176 | (0.039; 0.797) | ***0.024*** |
| **b2m** | 1.032 | (0.985; 1.081) | *0.191* |
| **CA** negative | 1.000 |  |  |
| **CA** positive | 0.250 | (0.055; 1.130) | *0.072* |
| **LDH** | 1.233 | (1.092; 1.393) | ***0.001*** |
| **CA** negative | 1.000 |  |  |
| **CA** positive | 0.204 | (0.046; 0.906) | ***0.037*** |
| **CRP** | 1.009 | (0.997; 1.022) | *0.138* |
| **CA** negative | 1.000 |  |  |
| **CA** positive | 0.179 | (0.041; 0.788) | ***0.023*** |
| **mIg** | 0.993 | (0.972; 1.014) | *0.508* |
| **CA** negative | 1.000 |  |  |
| **CA** positive | 0.198 | (0.044; 0.890) | ***0.035*** |
| **PC Infiltration** | 0.994 | (0.973; 1.014) | *0.540* |
| **CA** negative | 1.000 |  |  |
| **CA** positive | 0.212 | (0.060; 0.750) | ***0.016*** |
| **RB1 deletion** - negative | 1.000 |  |  |
| **RB1 deletion** - positive | 2.328 | (0.843; 6.430) | *0.103* |
| **CA** negative | 1.000 |  |  |
| **CA** positive | 0.244 | (0.068; 0.872) | ***0.030*** |
| **TP53 deletion** - negative | 1.000 |  |  |
| **TP53 deletion** - positive | 3.241 | (0.682; 15.396) | *0.139* |
| **CA** negative | 1.000 |  |  |
| **CA** positive | 0.235 | (0.065; 0.852) | ***0.028*** |
| **IgH disruption** - negative | 1.000 |  |  |
| **IgH disruption** - positive | 0.736 | (0.257; 2.107) | *0.568* |
|  |  |  |  |
| **CA** negative | 1.000 |  |  |
| **CA** positive | 0.298 | (0.081; 1.091) | *0.068* |
| **t(4;14)** - negative | 1.000 |  |  |
| **t(4;14)** - positive | 1.198 | (0.261; 5.487) | *0.816* |
| **CA** negative | 1.000 |  |  |
| **CA** positive | 0.220 | (0.062; 0.777) | ***0.019*** |
| **amp1q21** - negative | 1.000 |  |  |
| **amp1q21** - positive | 1.221 | (0.456; 3.266) | *0.691* |
| **CA** negative | 1.000 |  |  |
| **CA** positive | 0.229 | (0.064; 0.817) | ***0.023*** |
| **Ploidy** - NHRD-MM | 1.000 |  |  |
| **Ploidy** - HRD-MM | 0.767 | (0.271; 2.170) | *0.618* |
| *Event of interest is death (‘related to disease’ or ‘other death reason’)* | | | |
